# Molecular epidemiology of rotavirus A and adenovirus among children with acute diarrhea in Hangzhou, China

**DOI:** 10.1186/s13099-020-00359-4

**Published:** 2020-04-13

**Authors:** Wei Li, Wenqing Xiang, Cixiu Li, Jialu Xu, Dongming Zhou, Shiqiang Shang

**Affiliations:** 1 Department of Clinical Laboratory, Children’s Hospital, Zhejiang University School of Medicine; National Clinical Research Center For Child Health, 3333 Binsheng Road, Hangzhou, 310052 People’s Republic of China; 2 Marie Bashir Institute for Infectious Diseases and Biosecurity, Charles Perkins Centre, School of Life & Environmental Sciences and Sydney Medical School, The University of Sydney, Sydney, NSW 2006 Australia; 3 Department of Neurology, Children’s Hospital, Zhejiang University School of Medicine; National Clinical Research Center For Child Health, Hangzhou, People’s Republic of China

**Keywords:** Rotavirus A, Adenovirus, Molecular epidemiology, Children

## Abstract

**Background:**

Rotavirus A (RVA) and adenovirus (Adv) are important causes of acute diarrhea in children. RVAs are classified into G and P genotypes based on viral proteins (VP)7 and VP4 gene and Adv contains over 70 genotypes based on hexon and fiber gene. This study aimed to characterize the molecular epidemiology of RVA and Adv in children with acute diarrhea during 2017–2018 in Hangzhou.

**Methods:**

The stool samples were collected and tested for RVA and Adv by reverse transcription-quantitative PCR (RT-qPCR) assay. The RVA positive samples were detected by RT-PCR for VP7(G) and VP4([P]) genotypes, and the Adv positive samples were detected by PCR for genotyping by the target to hexon gene.

**Results:**

Among 228 RVA-positive samples, G9 was detected as the most frequent genotype (195/228, 85.5%), followed by G3 (20/228, 8.8%), G2 (7/228, 3.1%) and G1 (6/228, 2.6%). G9 strains were closely related to strains from China and neighboring countries, as well as the USA. On the other hand, P[8] strains were detected in 219 (96.1%) samples with most closely related to one strain from Malawi, and P[4] in 9 (3.9%) samples. G9P[8] (84.6%, 193/228) was the most prevalent rotavirus A strains, followed by G3P[8] (8.8%, 20/228), G2P[4] (3.1%, 7/228), G1P[8] (2.6%, 6/228) and G9P[4] (0.9%, 2/228). Of 167 Adv-positive cases, 2 different genotypes were identified with 152 (91.0%) of Adv-41and 15 (9%) of Adv-40. All Adv strains were closely related to prototype strains of Adv types 40 and 41 in India.

**Conclusions:**

G9P[8] of RVA and Adv-41 were the most common genotypes that caused children’s acute diarrhea in Hangzhou, 2017–2018.

## Introduction

Acute diarrhea is most commonly seen disease in young children with gastroenterological disorder and dehydration. Rotavirus A (RVA) and adenovirus (Adv) are the main viruses that cause acute diarrhea in children [1]. RVAs are non-enveloped, and double-stranded RNA viruses that belong to the Reoviridae family. According to viral proteins (VP)7 and VP4, the RVAs are classified into G and P genotypes [2]. Currently, there are 27 G-genotypes, 35 P-genotypes, and at least 73 G/P-genotype combinations existed. Genotypes G1–G4 and G9 are reported to be the most commonly detected G-genotypes among the children worldwide [3]. Distribution of different combinations of RVA genotypes varied both geographically and chronologically. According to a previous study, G1P[8], G2P[4], G3P[8], G4P[8] and G9P[8] are regarded as the common genotypic combinations throughout the world [4].

Adenovirus is a linear, double-stranded DNA virus with a genome size of 26–45 kb. Over 70 genotypes in seven species (Adv A–G) have been characterized and classified phylogenetically according to the nucleic acid characteristics and homology as well as their hexon and fiber protein characteristics [5]. Among these species, Adv types, especially Adv-40 and -41 were found to frequently cause diarrhea in children [6]. In this study, the molecular characteristics of RVA and Adv in children in Hangzhou upon the onset of viral diarrhea were evaluated.

## Methods

### Study population

In this study, stool sample was collected from each child who visited Children’s Hospital of Zhejiang University School of Medicine in the inpatient wards and outpatient departments and was diagnosed as acute diarrhea with suspected virus infections. According to the manufacturer’s instruction, all stool samples were performed rapid antigen testing (Abon Biopharm Company, Hangzhou, China; CFDA No.: 20153402309) within 2 h after stool sample collected. The rapid antigen testing positive samples were frozen at − 80 °C for further using. The rapid antigen testing positive stool samples were randomly selected and performed for RVA and/or Adv nucleic acid detection by reverse transcription-quantitative PCR (RT-qPCR) assay (Wokang biotech, China). This study was approved by the medical ethics committee of the Children’s Hospital of Zhejiang University School of Medicine (NO.2018-IEC-001).

### Detection of rotavirus A or/and adenovirus

Stools samples were collected and mixed with 1 ml normal saline. The mixtures were centrifuged at 6000 rpm at 20 °C for 30 s. 200 μl supernatant was separated and DNA/RNA was extracted by NAE32 nucleic acid automatic extraction instrument (Daan gene, China). The one step RT-qPCR amplification was performed in a total volume of 25 μl with the SLAN 96P real time PCR System (HONGSHI, China). The reaction mixtures consisted of 5 μl sample and 20 μl one step RT-qPCR reagent (wokang biotech, China), respectively. Each run under the following conditions: 20 min at 50 °C; 5 min at 95 °C; 5 cycles of 10 s at 95 °C, 15 s at 55 °C and 30 s at 72 °C; And then followed by 35 cycles of 10 s at 95 °C and 45 s at 60 °C. FAM channel was used to detect rotavirus A and HEX channel was performed to detect adenovirus. Template of one step RT-qPCR positive sample was used for further genotyping of RVA or Adv. According to manufacturer’s instructions, the sensitivity of this commercial kit was at least of 1.0 × 10^3^ PFU/ml, and this assay was negative for detection of other gut pathogens, such as norovirus, astrovirus, enterovirus, Salmonella, Escherichia coli and etc.

### Genotyping of rotavirus A and adenovirus

The amplification of RVA VP7 and VP4 gene was performed by using 960 PCR instrument (Heal Force, China) through one-step RT–PCR assay. One-step RT–PCR assay reagents and primers were supported by Chongqing Wokang Biotechnology Co., Ltd. The forward primer of VP7 gene: 5′ GAATCAAATAARTGGATATCAATGGG 3′, and reverse primer of VP7 gene was 5′GCTACRTTTTCCCTYGGTCC3′. The forward primer of VP4 gene was 5′TTTACACCACCCAMTGATTATTGG3′, and reverse primer of VP4 gene was 5′ CTCTAAACGTTTCGAAAAAYTTCCA3′. One step RT-PCR was conducted under the following conditions: 20 min at 50 °C; 5 min at 95 °C; 5 cycles of 10 s at 95 °C, 15 s at 55 °C and 30 s at 72 °C, followed by 35 cycles of 10 s at 95 °C and 45 s at 60 °C. The amplification of Adv hexon gene underwent genotyping. The primers were as follows: Forward primer: 5′ GGTCTTACATGCACATCGCC3′; and Reverse primer: 5′ CAAAACCCGGTTGTCGCC 3′. PCR assay was conducted under the following conditions: 5 min at 95 °C; 5 cycles of 10 s at 95 °C, 15 s at 55 °C and 30 s at 72 °C, and followed by 35 cycles of 10 s at 95 °C and 45 s at 60 °C. The amplification products were purified and sequenced in the Generay company (Shanghai, China). Sequences of RVA or Adv isolates were compared to the National Center for Biotechnology Information (NCBI) database through BLAST. Nucleotide sequences of representative strains were compared with reference strains of each virus using Mafft alignment with L-INS-I algorithm. Phylogenetic analysis was performed by maximum likelihood method that is implemented in the program PhyML 3.0 with GTR model.

### Statistical analysis

The results were analyzed using SPSS software (version 20.0). χ^2^ test was used to analyze statistical differences. Two-tailed P values of less than 0.05 were considered to be statistically significant.

## Results

### Genotyping of rotavirus A

**Fig. 1:**
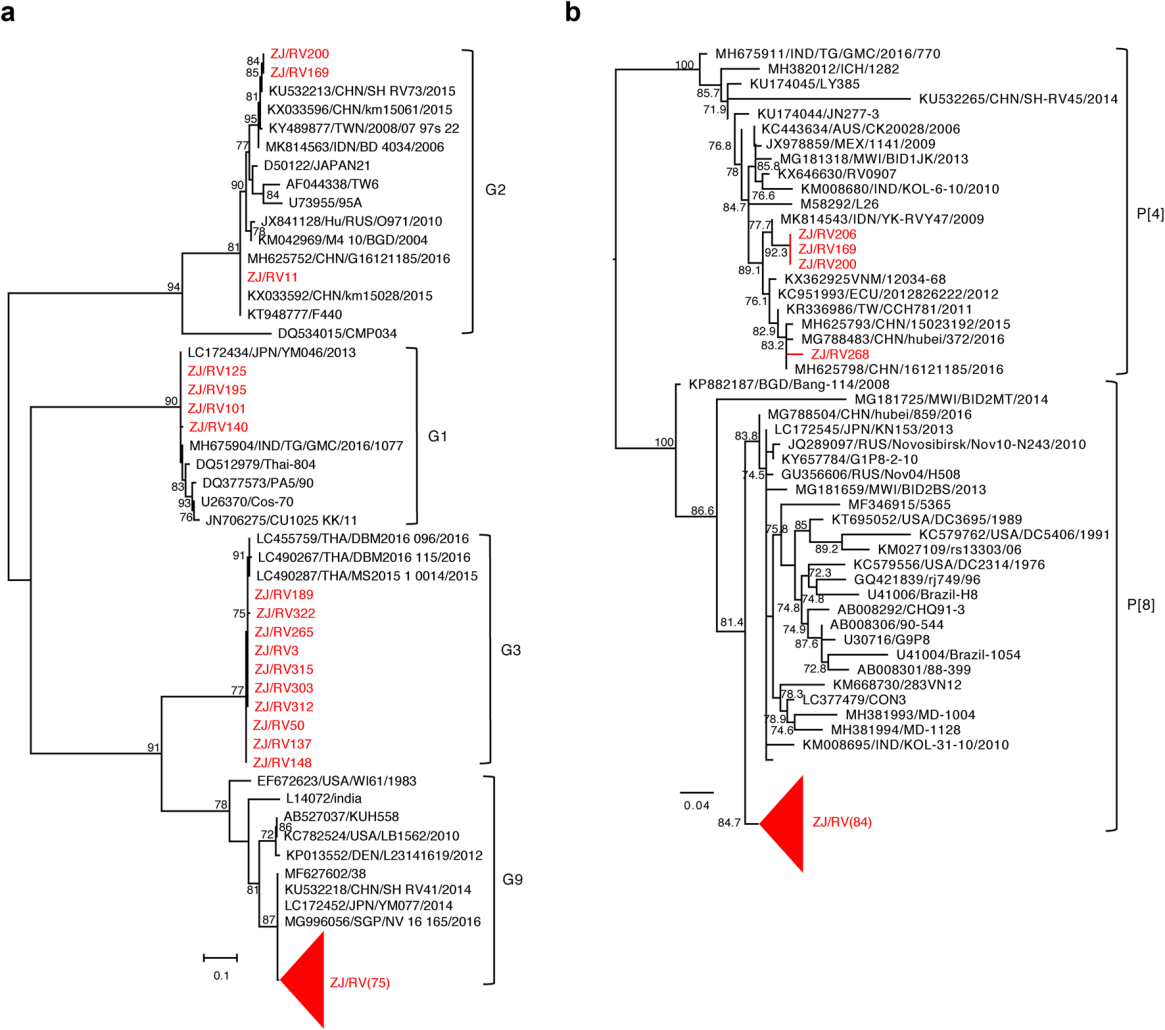
Maximum-likelihood (ML) phylogenetic tree based on a portion of VP4 or VP7 gene of rotavirus A. **a** G gene. **b** P gene. Bootstrap values greater than 70% are indicated

In study period, 35,871 samples were performed rapid antigen testing, 6196 (17.3%) were positive for RVA and 1112 (3.1%) were positive for Adv. The genotyped samples were randomly selected throughout the year of 2017–2018. To ensure that these samples were representative, we collect a certain number of samples according to the positive rate of each month in 2017–2018. A total of 228 RVA-positive samples were collected for this study. To determine the subtype of each RVA, the VP4 and VP7 genes of RVAs were amplified and then sequenced. Phylogenetic trees were constructed from the nucleotide sequences of partial VP4 and VP7 genes. Among the 228 RVA-positive cases, only 4 different RVA G genotypes were detected. Of these, G9 (85.5%, 195/228) was the most frequently detected genotype (*P *< 0.05), followed by G3 (8.8%, 20/228), G2 (3.1%, 7/228) and G1 (2.6%, 6/228). As shown in Fig. 1a, G2 strains detected in this study showed close association with strains of China, and G3 strains showed close association with strains of Thailand, while G1 and G9 strains showed close association with those of Japan, India, Thailand, USA as well as China. For genotype of P gene, 2 different P gene genotypes, including 219 of P[8] (96.1%, 219/228) and 9 of P[4] (3.9%, 9/228) were detected, and RVA of P[8] was significantly higher than P[4] (*P *< 0.05). Phylogenetic analyses (Fig. 1b) detected P[4] strains that are highly identical with those of China and Indonesia. However, all P[8] strains were clustered into a lineage, and showed close relation to one of the strains of Malawi (MG181725/MWI/BID2MT/2014). Simultaneous analysis of VP7 gene and VP4 gene revealed G9P[8] (84.6%, 193/228) as the most prevalent rotavirus strains that are associated with acute diarrhea in Hangzhou children (*P *< 0.05), followed by G3P[8] (8.8%, 20/228), G2P[4] (3.1%, 7/228), G1P[8] (2.6%, 6/228) and G9P[4] (0.9%, 2/228).

### Genotyping of adenovirus

**Fig. 2:**
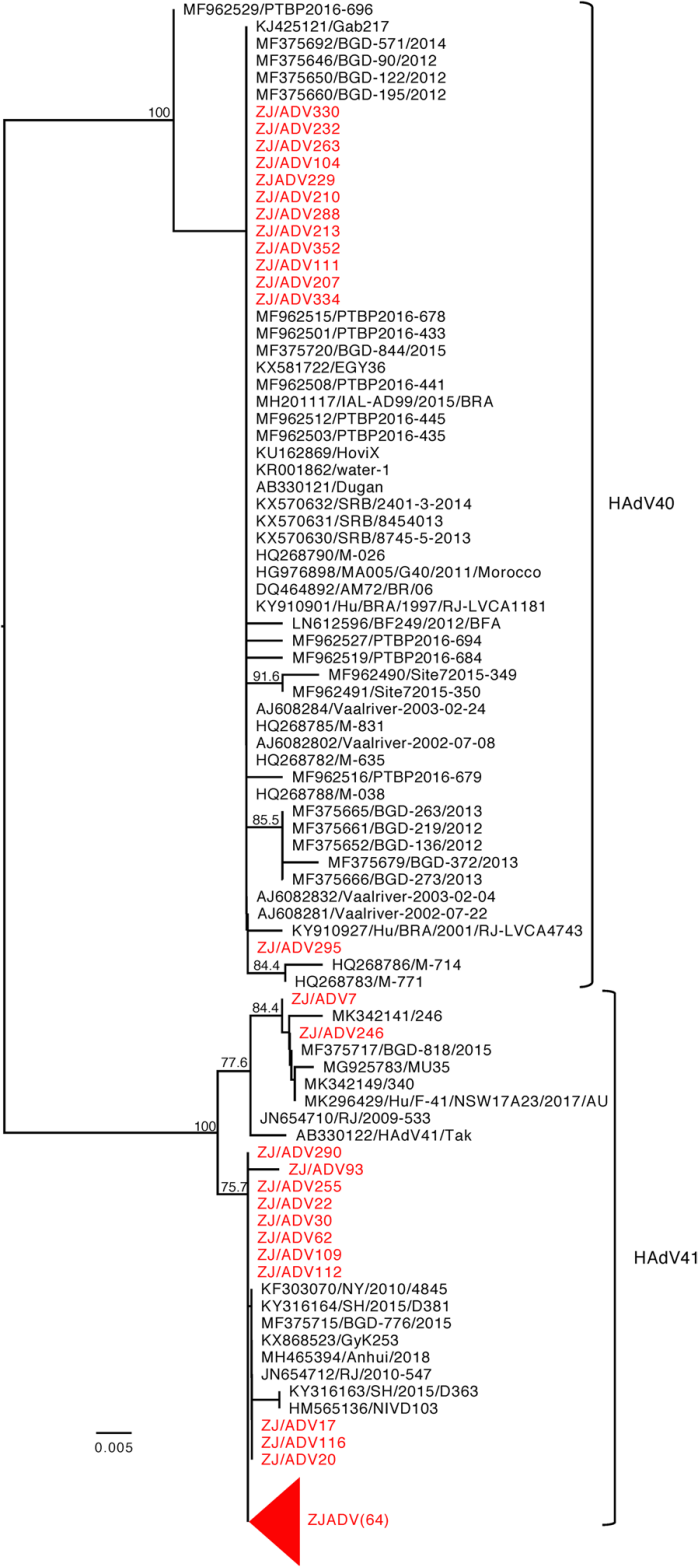
Maximum-likelihood (ML) phylogenetic tree based on a portion of hexon gene of adenovirus. Bootstrap values greater than 70% are indicated

During the study period, 167 Adv-positive samples were also randomly collected and genotyped. Of the 167 Adv-positive samples, 2 different genotypes were identified. Adv-41 (91.0%, 152/167) was considered to be the most frequent genotype found in Hangzhou that was significantly higher than Adv-40 (9.0%, 15/167, *P *< *0.05*). As shown in Fig. 2, phylogenetic tree was constructed based on partial hexon gene when compared to the previously reported strains, including the prototype strains of Adv types 40 and 41 in India. Adv type 40 that was detected in this study exhibited a similarity of greater than 99% with the prototype strain Dugan, while Adv type 41 exhibited more than 98% similarity with the prototype strain Tak.

## Discussion

Before 2010, G1 and G3 were considered as predominant rotaviruses circulating alternately around China [7–11]. After 2010, G9 began to increase and became the main epidemic subtype in the mainland of China, with a proportion of 91.8% in eastern China in 2013–2014 [12], 66.4% in southwest China in 2014–2015 [13], and 79.4% in Kunming in 2015–2016 [14]. Furthermore, some reports were inconsistent with these surveillance results, as G3 was the predominant subtype in Qinghai province in 2012–2015, and G1 was the major subtype in Henan province in 2009–2015 [15, 16]. However, there were fewer reports that reported about G surveillance results in recent years. In this study, our surveillance also suggested G9 as the most prevalent genotype in Hangzhou in 2017–2018. Also G9 strains showed close association with those of China and neighboring countries, as well as the USA. According to the previous study, P[8] and P[4] RVAs have been identified as the most common P-genotypes worldwide [2]. In our study, 2 different P gene genotypes including P[8] and P[4] were found, and P[8] (96.1%) of these was the most common genotype in Hangzhou in 2017–2018. Phylogenetic tree analysis revealed that the P[8] strains showed close association to one of the strains from Malawi. By combining VP4 and VP7 results, G9P[8] (84.6%) was regarded as the most common strain that caused acute gastroenteritis in Hangzhou children. The proportion of G1P[8] and G3P[8] was less than 10%, and these genotypes were also common before 2010. Compared with other studies in different areas of China, our results also supported G9P[8] as the major genotype of RVA in recent years [12–14, 17]. In the future, more research should be conducted to confirm whether G9P[8] is the most popular strain in China.

In our surveillance, Adv-41 (91.0%) was found to be the most frequent genotype infected in children in Hangzhou in 2017–2018. These results were similar to that of previous studies, wherein Adv-41 was predominant adenovirus genotype identified in Shanghai, China, 2006 [18], 92.1% of Adv41 was detected in Brazil in 2012–2017 [19], and Adv41, 40 and Adv3 were identified as the most dominant ones in Hebei, China in 2017 [20]. Adv type 40 strains showed close association to the prototype strain Dugan, while Adv type 41 strains exhibited significant similarity with the prototype strain Tak [21]. Recently, the proportion of Adv-3 was increasing in children with diarrhea in China [19, 21]. However, Adv-3 was not detected, which is a limitation in our study. The surveillance of Adv-3 in Hangzhou, China should be strengthened in the future.

In conclusion, G9, P[8] and G9P[8] were the most common genotypes of RVA strains, and Adv-41 was a prevalent Adv that caused acute diarrhea in children of Hangzhou in 2017–2018. The survey of RVA genotype and adenovirus from children with acute diarrhea warrants further research.

## Data Availability

All the data are within the manuscript.
